# Supplementary material for: Laboratory evolution of synthetic electron transport system variants reveals a larger metabolic respiratory system and its plasticity
Source: Nat Commun. 2022 Jun 27;13:3682. doi: 10.1038/s41467-022-30877-5 (PMC9237125; doi:10.1038/s41467-022-30877-5)
Supplement: Supplementary file 1 — Supplementary information [file 41467_2022_30877_MOESM1_ESM.pdf]

Supplementary Information

Contents:

Supplementary Tables (4)

Supplementary Figure (4)

Details of supplementary data

Supplementary Table 1: Sample code used during running the ALE machine

**Strain**	**ALE replicate number**	**Full ALE code**
*u*ETS-1H	Unevolved	Δ*nuoB*Δ*cyoB*_A0F0I1R
*e*ETS-1HA	ALE-1	Δ*nuoB*Δ*cyoB*_A9F63I1R1
*e*ETS-1HB	ALE-2	Δ*nuoB*Δ*cyoB*_A10F64I1R1
*e*ETS-1HC	ALE-3	Δ*nuoB*Δ*cyoB*_A11F64I1R1
*e*ETS-1HD	ALE-4	Δ*nuoB*Δ*cyoB*_A12F63I1R1
*u*ETS-2H	Unevolved	Δ*nuoB*Δ*cydB*Δ*appC*_A0F0I1R1
*e*ETS-2HA	ALE-1	Δ*nuoB*Δ*cydB*Δ*appC*_A13F59I1R1
*e*ETS-2HB	ALE-2	Δ*nuoB*Δ*cydB*Δ*appC*_A14F58I1R1
*e*ETS-2HC	ALE-3	Δ*nuoB*Δ*cydB*Δ*appC*_A15F57I1R1
*e*ETS-2HD	ALE-4	Δ*nuoB*Δ*cydB*Δ*appC*_A16F57I1R1
*u*ETS-3H	Unevolved	Δ*ndh*Δ*cyoB*_A0F0I1R1
*e*ETS-3HA	ALE-1	Δ*ndh*Δ*cyoB*_A5F59I1R1
*e*ETS-3HB	ALE-2	Δ*ndh*Δ*cyoB*_A6F58I1R1
*e*ETS-3HC	ALE-3	Δ*ndh*Δ*cyoB*_A7F56I1R1
*e*ETS-3HD	ALE-4	Δ*ndh*Δ*cyoB*_A8F59I1R1
*u*ETS-4H	Unevolved	Δ*ndh*Δ*cydB*Δapp*C*_A0F0I1R1
*e*ETS-4HA	ALE-1	Δ*ndh*Δ*cydB*Δapp*C*_A9F66I1R1
*e*ETS-4HB	ALE-2	Δ*ndh*Δ*cydB*Δapp*C*_A10F71I1R1
*e*ETS-4HC	ALE-3	Δ*ndh*Δ*cydB*Δapp*C*_A11F63I1R1
*e*ETS-4HD	ALE-4	Δ*ndh*Δ*cydB*Δapp*C*_A12F60I1R1

Supplementary Table 2: Key genetic changes in evolved ETS variants

Strain	Media adaptation-related mutations	Condition-specific convergent gene mutation
*e*ETS-1HA	+5 bp between *hns* and *tdk* at genomic position 1293009, Δ82 bp between *pyrE* and *rph* at genomic position 3815859	None
*e*ETS-1HB	+5 bp between *hns* and *tdk* at genomic position 1293009, Δ82 bp between *pyrE* and *rph* at genomic position 3815859	None
*e*ETS-1HC	G to T substitution in *rpoC* at genomic position 4188513, Δ82 bp between *pyrE* and *rph* at genomic position 3815859	None
*e*ETS-1HD	+5 bp between *hns* and *tdk* at genomic position 1292997, Δ82 bp between *pyrE* and *rph* at genomic position 3815859	None
*e*ETS-2HA	A to T substitution in *rpoC* at genomic position 4187214	None
*e*ETS-2HB	C to T substitution in *rpoC* at genomic position 4185540	None
*e*ETS-2HC	A to G substitution in *rpoC* at genomic position 4187214	None
*e*ETS-2HD	C to T substitution in *rpoC* at genomic position 4185540	None
*e*ETS-3HA	Δ82 bp between *pyrE* and *rph* at genomic position 3815859	T to C substitution in *sdhA* at genomic position 756099
*e*ETS-3HB	Δ82 bp between *pyrE* and *rph* at genomic position 3815859	C to T substitution in *sdhA* at genomic position 756150
*e*ETS-3HC	Δ82 bp between *pyrE* and *rph* at genomic position 3815859	T to G substitution in *sdhA* at genomic position 756886
*e*ETS-3HD	Δ82 bp between *pyrE* and *rph* at genomic position 3815859	C to G substitution in *sdhA* at genomic position 756968
*e*ETS-4HA	C to A substitution in *rpoA* at genomic position 3440923	C to T substitution in *yjjX* at genomic position 4633743
*e*ETS-4HB	Δ3 bp in *rpoB* at genomic position 4183399	C to T substitution in *yjjX* at genomic position 4633634
*e*ETS-4HC	A to G substitution in *rpoC* at genomic position 4186578	C to T substitution in *yjjX* at genomic position 4633657
*e*ETS-4HD	C to T substitution in *rpoC* at genomic position 4189448	C to T substitution in *yjjX* at genomic position 4633657

The intergenic mutation between *pyrE* and *rph* was present in the respective unevolved strain itself and these are listed here for a better context of media adaptation. Other mutations present in unevolved strain have not been listed here. The genes mutated in all four evolved replicates of a strain are listed here as convergent gene mutations. A complete list of every genetic change can be found in the Supplementary data 1.

Supplementary Table 3: Phenotypic characterization of the strains of the study

Strain	Growth rate (h^{-1})	Glucose uptake rate (mmol/gDCW/h)	Acetate secretion rate (mmol/gDCW/h)
*u*ETS-1H	0.76, 0.79	12.77, 13.1	12.01, 12.35
*e*ETS-1HA	0.88, 0.9	13.57, 14.24	13.14, 13.61
*e*ETS-1HB	0.87, 0.82	13.61, 13.23	13.03, 12.88
*e*ETS-1HC	0.83, 0.82	13.84, 13.96	13.24, 12.93
*e*ETS-1HD	0.83, 0.83	13.35, 13.77	12.64, 13.06
*u*ETS-2H	0.65, 0.64	9.51, 9.26	6.41, 6.08
*e*ETS-2HA	0.87, 0.92	11.24, 11.71	7.76, 7.92
*e*ETS-2HB	0.9, 0.88	11.57, 11.67	7.77, 7.4
*e*ETS-2HC	0.9, 0.9	11.62, 11.68	7.36, 7.53
*e*ETS-2HD	0.89, 0.89	11.65, 11.76	7.98, 7.85
*u*ETS-3H	0.42, 0.43	7.68, 8.02	6.67, 6.51
*e*ETS-3HA	0.82, 0.83	12.6, 12.58	11.03, 11.26
*e*ETS-3HB	0.82, 0.86	12.35, 12.72	11.18, 11.26
*e*ETS-3HC	0.83, 0.84	12.78, 12.87	11.27, 10.97
*e*ETS-3HD	0.83, 0.84	11.92, 12.26	10.81, 11.07
*u*ETS-4H	0.53, 0.52	7.96, 8.18	3.95, 3.81
*e*ETS-4HA	0.85, 0.84	9.4, 9.01	4.24, 3.77
*e*ETS-4HB	0.86, 0.88	10.06, 10.47	5.25, 6.22
*e*ETS-4HC	0.79, 0.83	9.2, 9.3	6.14, 5.97
*e*ETS-4HD	0.8, 0.82	9.32, 9.49	4.96, 4.84

The values for two independent replicates are listed in the table.The levels of Succinate, Lactate, Formate, Ethanol, and Pyruvate were below the detection limit.

Supplementary Table 4: Description of the ATS iModulons

S. No.	iModulon	Genes (negative coefficients in red)
1	iModulon-13	*sdhB, cyoD, sdhD, cyoA, sdhA, sdhC, cyoC, fumA*
2	iModulon-b2287	*nuoB*
3	iModulon-8	*ndh, cyoB*
4	iModulon-11	*cydB, appB, cyoB, appC, ndh*
5	iModulon-b3366	*nirD*
6	iModulon-7	*nirB, grcA*
7	iModulon-2	*narI, narJ*
8	iModulon-1	*yliI, cyoD*
9	iModulon-16	*hyaB, hyaA, hyaC*
10	iModulon-9	*hybO, hybA*
11	iModulon-10	*tktB, adhP, fbaB, talA, poxB*

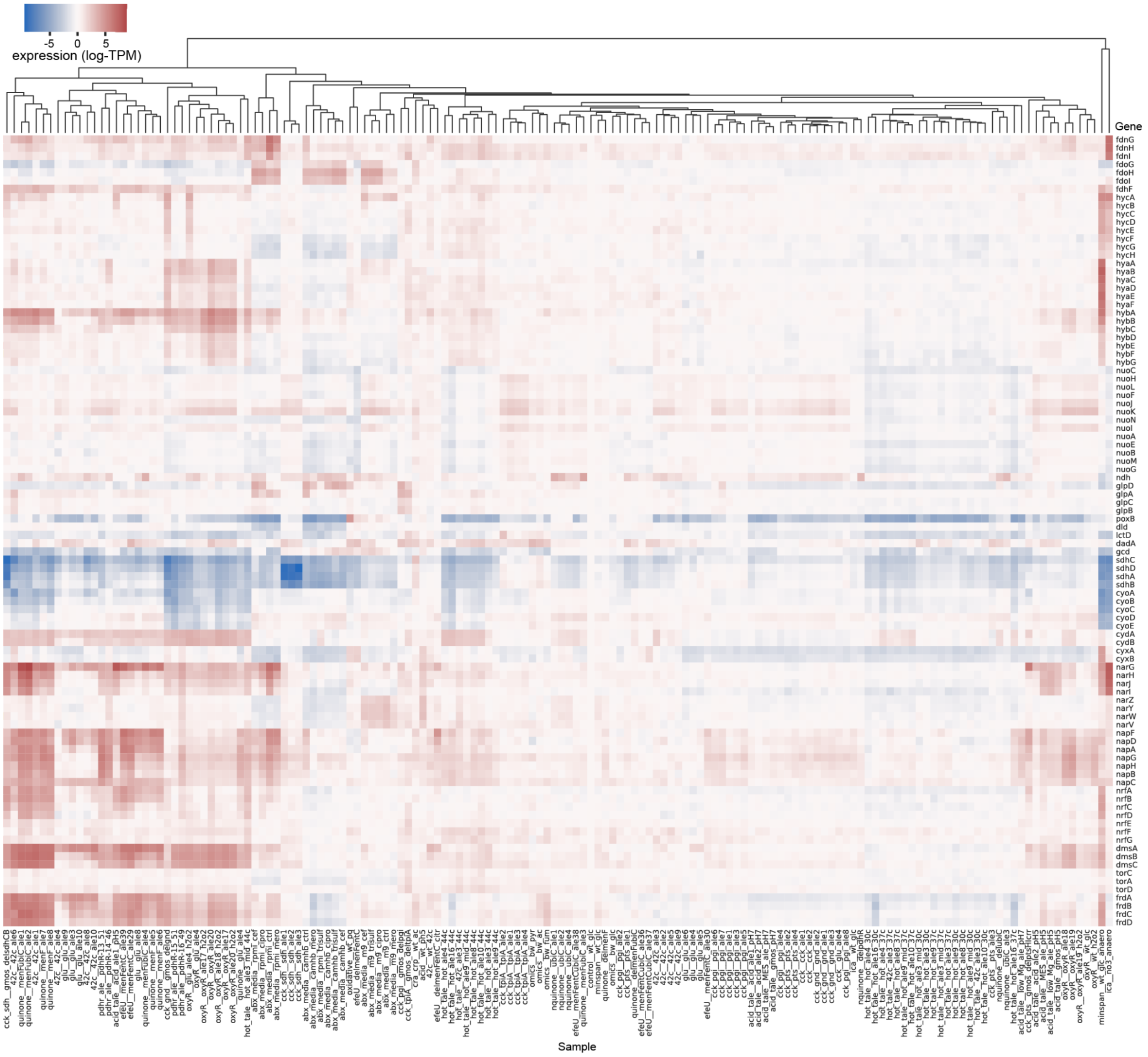

Supplementary Figure 1: Expression levels of respiratory dehydrogenase and reductases of *E. coli*. The entire RNA-seq compendium was explored and the conditions directly or indirectly associated with energy metabolism have been displayed.

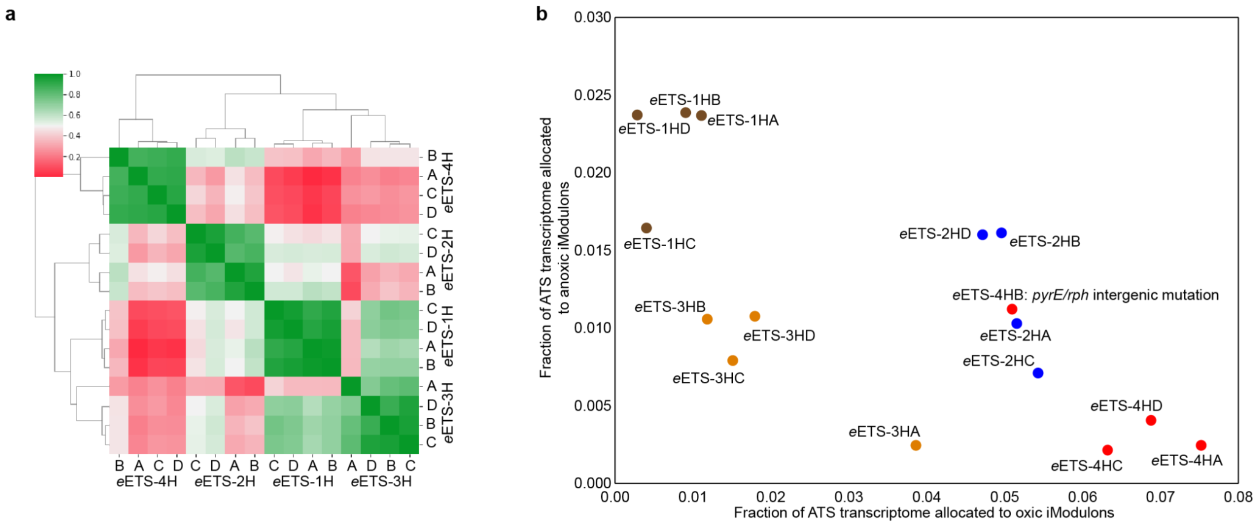

Supplementary Figure 2: (a) Correlation among ETS variants based on the ATS iModulon activities. (b) The enlarged representation of figure 3d lists the replicate information and potential genetic change in the outlier replicate *e*ETS-4HB.

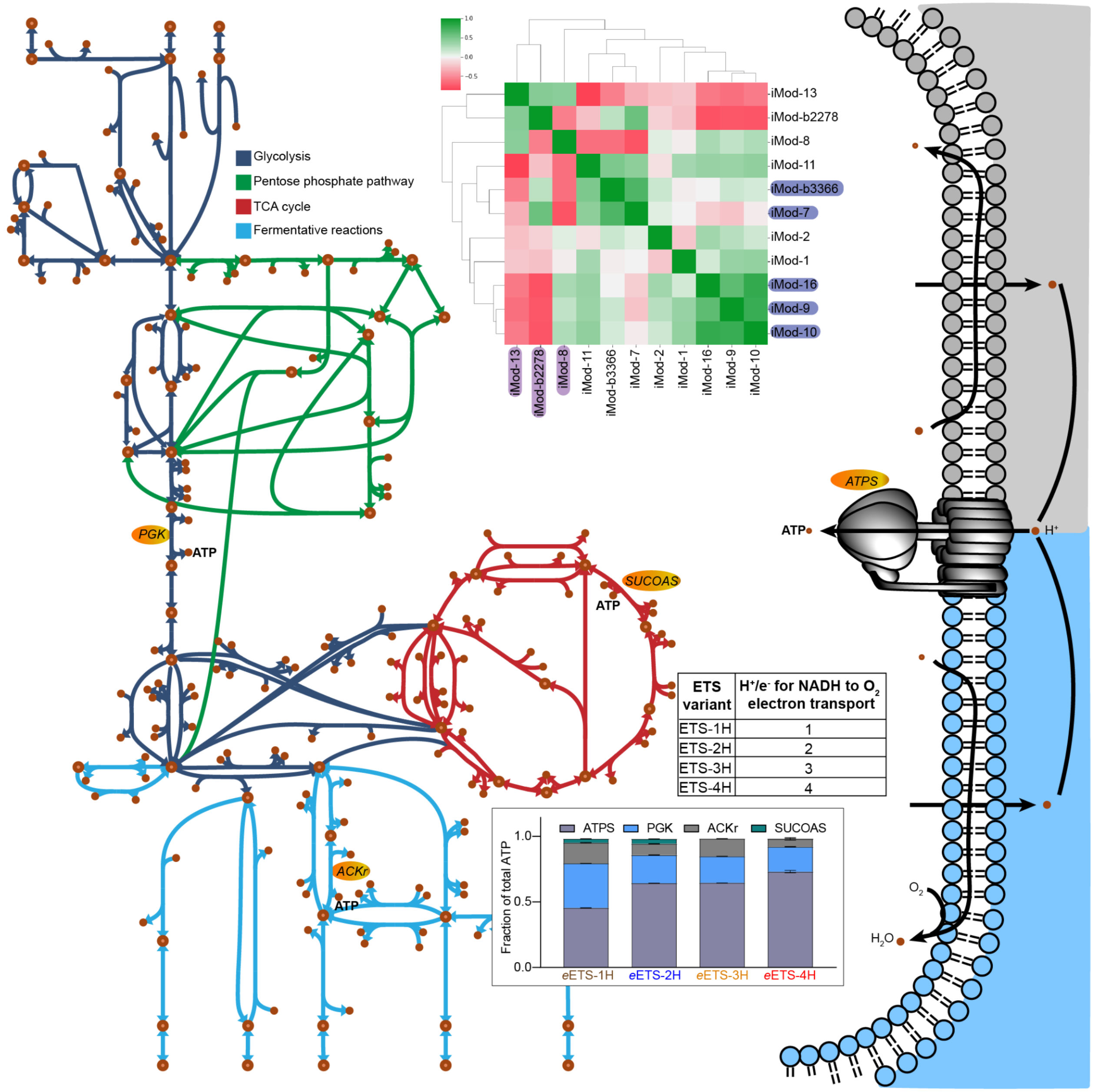

Supplementary Figure 3: Scheme of Aero-type system (ATS). The pathways are showing reactions of ATS and major ATP production sites. Heatmap represents the correlation between the iModulons within ATS. iModulons showing a tradeoff are highlighted. The histogram from Figure 3 has been included to show adjustments within ATS to achieve a similar ATP yield. Part of the membrane highlighted in blue represents oxic ETS and another part highlighted in gray represents anoxic ETS. The list of ATS genes was generated based on COG and GO categories to include as many relevant genes as possible to represent pathways involved in ATP production, then filtered to remove genes that are never expressed in the multiple model simulations.

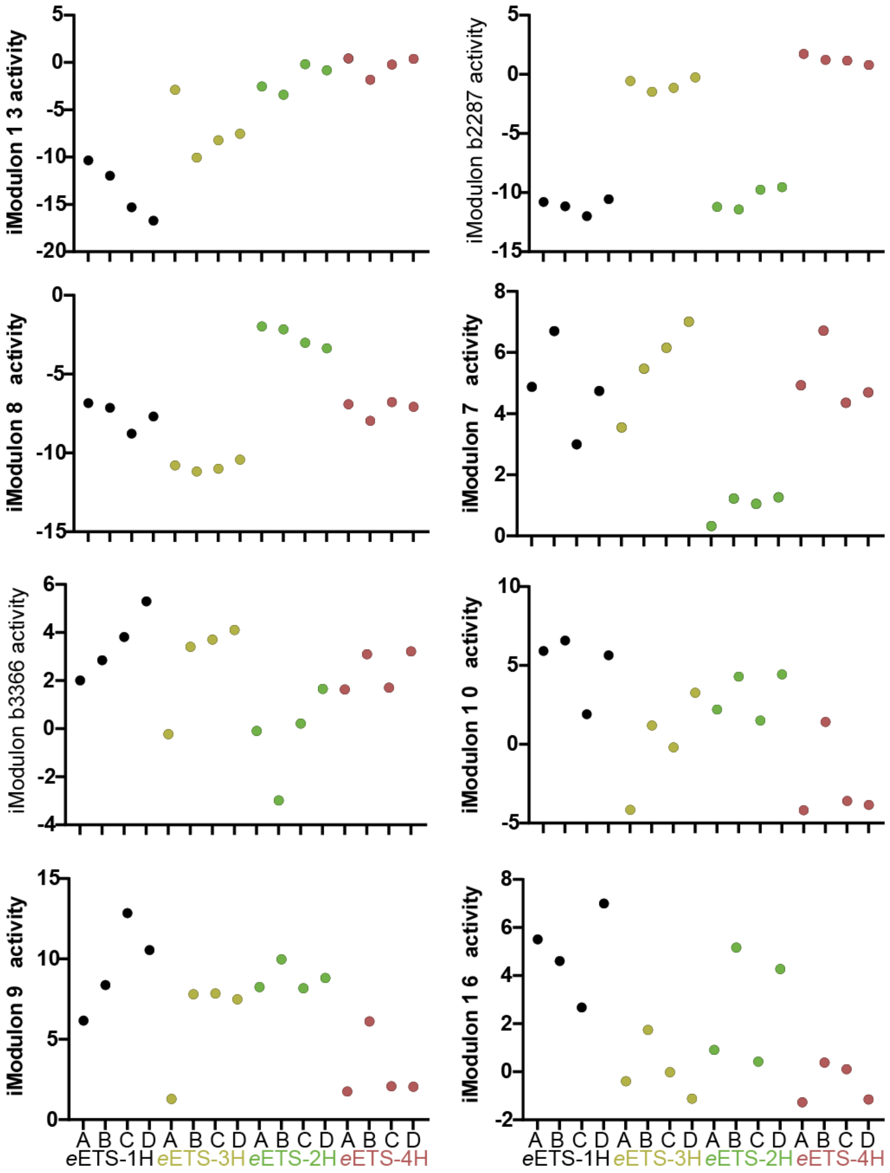

Supplementary Figure 4: Activities of ATS iModulons in the unevolved and evolved ETS variants calculated using independent component analysis. The gene membership of corresponding iModulons is listed in Supplementary Table 3.

Details of supplementary data

Supplementary data 1 presents the complete list of the mutations observed in every strain of this study.

Supplementary data 2 presents the complete detail of the value of reaction flux for every strain of this study.

Supplementary data 3 presents the list of genes constituting the Aero-Type System.
