# Supplementary material for: Laboratory evolution of synthetic electron transport system variants reveals a larger metabolic respiratory system and its plasticity
Source: Nat Commun. 2022 Jun 27;13:3682. doi: 10.1038/s41467-022-30877-5 (PMC9237125; doi:10.1038/s41467-022-30877-5)
Supplement: Supplementary file 2 — Reporting Summary [file 41467_2022_30877_MOESM2_ESM.pdf]

nature portfolio

Corresponding author(s): Bernhard O. Palsson, Amitesh Anand

Last updated by author(s): May 12, 2022

Reporting Summary

Nature Portfolio wishes to improve the reproducibility of the work that we publish. This form provides structure for consistency and transparency in reporting. For further information on Nature Portfolio policies, see our Editorial Policies and the Editorial Policy Checklist.

Statistics

For all statistical analyses, confirm that the following items are present in the figure legend, table legend, main text, or Methods section.

n/a	Confirmed	
☐	☒	The exact sample size (*n*) for each experimental group/condition, given as a discrete number and unit of measurement
☐	☒	A statement on whether measurements were taken from distinct samples or whether the same sample was measured repeatedly
☒	☐	The statistical test(s) used AND whether they are one- or two-sided<br>*Only common tests should be described solely by name; describe more complex techniques in the Methods section.*
☒	☐	A description of all covariates tested
☒	☐	A description of any assumptions or corrections, such as tests of normality and adjustment for multiple comparisons
☐	☒	A full description of the statistical parameters including central tendency (e.g. means) or other basic estimates (e.g. regression coefficient) AND variation (e.g. standard deviation) or associated estimates of uncertainty (e.g. confidence intervals)
☒	☐	For null hypothesis testing, the test statistic (e.g. *F*, *t*, *r*) with confidence intervals, effect sizes, degrees of freedom and *P* value noted<br>*Give P values as exact values whenever suitable.*
☒	☐	For Bayesian analysis, information on the choice of priors and Markov chain Monte Carlo settings
☒	☐	For hierarchical and complex designs, identification of the appropriate level for tests and full reporting of outcomes
☒	☐	Estimates of effect sizes (e.g. Cohen's *d*, Pearson's *r*), indicating how they were calculated

Our web collection on statistics for biologists contains articles on many of the points above.

Software and code

Policy information about availability of computer code

Data collection	No software was used
Data analysis	The software scripts supporting the prediction of mutation effects to the encoding of genes described in this article are available in the following open-access archive repository: https://doi.org/10.5281/zenodo.5431595. All the simulations performed in this manuscript can be reproduced using the FoldME model, which is constructed using the COBRApy toolbox version 0.5.11 for constraint-based modeling and its extension for ME-models, COBRAme version 0.0.9, ECOLIme version 0.0.9, and solveME, all publicly available on Github (https://github/SBRG/cobrame/, https://github.com/SBRG/ecolime, https://github.com/SBRG/solvemepy). Custom code for constraining and solving ME-models can be found at https://github.com/SBRG/ME-script. GraphPad Prism version 9.2.0 was used for generating the plots.

For manuscripts utilizing custom algorithms or software that are central to the research but not yet described in published literature, software must be made available to editors and reviewers. We strongly encourage code deposition in a community repository (e.g. GitHub). See the Nature Portfolio guidelines for submitting code & software for further information.

Data

Policy information about availability of data

All manuscripts must include a data availability statement. This statement should provide the following information, where applicable:

- Accession codes, unique identifiers, or web links for publicly available datasets
- A description of any restrictions on data availability
- For clinical datasets or third party data, please ensure that the statement adheres to our policy

Resequencing and expression profiling data that support the findings of this study can be accessed from NCBI Sequence Read Archive (PRJNA835443) and Gene Expression Omnibus (GSE202144) respectively. The PRECISE compendium and all associated data files can be found at https://github.com/SBRG/precise2.and Gene

Expression Omnibus (GSE202144) respectively. aledb.org is the database of mutations from ALE experiments that have been referred to in this manuscript.

Field-specific reporting

Please select the one below that is the best fit for your research. If you are not sure, read the appropriate sections before making your selection.

- [x] Life sciences
- [ ] Behavioural & social sciences
- [ ] Ecological, evolutionary & environmental sciences

For a reference copy of the document with all sections, see nature.com/documents/nr-reporting-summary-flat.pdf

Life sciences study design

All studies must disclose on these points even when the disclosure is negative.

Sample size	No statistical methods were used to predetermine sample sizes. Laboratory evolution experiments were performed with four independent replicates which is a standard practice to effectively run the Adaptive Laboratory Evolution platform. Example: PMID: 33826886, PMID: 31767748.
Data exclusions	No exclusion
Replication	Four independent biological replicates were used for the adaptive laboratory evolution experiments and reproducible data were obtained for each replicate. RNA expression and phenotype characterization data were generated in duplicate and each replicate has been reported independently.
Randomization	Experimental group allocation was not needed
Blinding	No such group allocation was required in this study

Reporting for specific materials, systems and methods

We require information from authors about some types of materials, experimental systems and methods used in many studies. Here, indicate whether each material, system or method listed is relevant to your study. If you are not sure if a list item applies to your research, read the appropriate section before selecting a response.

Materials & experimental systems

n/a	Involved in the study	
[x]	[ ]	Antibodies
[x]	[ ]	Eukaryotic cell lines
[x]	[ ]	Palaeontology and archaeology
[x]	[ ]	Animals and other organisms
[x]	[ ]	Human research participants
[x]	[ ]	Clinical data
[x]	[ ]	Dual use research of concern

Methods

n/a	Involved in the study	
[x]	[ ]	ChIP-seq
[x]	[ ]	Flow cytometry
[x]	[ ]	MRI-based neuroimaging
