# Supplementary material for: Laboratory evolution of synthetic electron transport system variants reveals a larger metabolic respiratory system and its plasticity
Source: Nat Commun. 2022 Jun 27;13:3682. doi: 10.1038/s41467-022-30877-5 (PMC9237125; doi:10.1038/s41467-022-30877-5)
Supplement: Supplementary file 4 — Description of Additional Supplementary Files [file 41467_2022_30877_MOESM4_ESM.pdf]

Title: Supplementary data 1
Description: presents the complete list of the mutations observed in strains described in this study.

Title: Supplementary data 2
Description: presents the complete detail of the value of reaction flux for every strain of this study.

Title: Supplementary data 3
Description: presents the list of genes constituting the Aero-Type System.
